# Antibacterial Activity of Peptide Derivatives of Phosphinothricin against Multidrug-Resistant *Klebsiella pneumoniae*

**DOI:** 10.3390/molecules28031234

**Published:** 2023-01-27

**Authors:** Marija V. Demiankova, Fabio Giovannercole, Maxim A. Khomutov, Arthur I. Salikhov, Laura Onillon, Vladimir T. Valuev-Elliston, Byazilya F. Vasilieva, Elena N. Khurs, Nina I. Gabrielyan, Sergey N. Kochetkov, Olga V. Efremenkova, Daniela De Biase, Alex R. Khomutov

**Affiliations:** 1 Gause Institute of New Antibiotics, Bol’shaya Pirogovskaya 11, 119021 Moscow, Russia; 2 Department of Medico-Surgical Sciences and Biotechnologies, Sapienza University of Rome, Corso della Repubblica 79, I-04100 Latina, Italy; 3 Engelhardt Institute of Molecular Biology, Russian Academy of Sciences, Vavilov Street 32, 119991 Moscow, Russia; 4 Academician V.I. Shumakov Federal Research Centre of Transplantology and Artificial Organs, 123182 Moscow, Russia

**Keywords:** multidrug-resistant bacteria, *Klebsiella pneumoniae*, *Escherichia coli*, glutamine synthetase, phosphinothricin, Bialaphos

## Abstract

The fast spread of bacteria that are resistant to many classes of antibiotics (multidrug resistant) is a global threat to human and animal health with a worrisome scenario ahead. Novel therapeutical strategies are of crucial importance to combat this phenomenon. For this purpose, we investigated the antimicrobial properties of the naturally occurring tripeptide Bialaphos and a dipeptide *L*-leucyl-*L*-phosphinoithricin, the synthesis and diastereomers separation of which are herein described. We demonstrate that these compounds are effective on clinical isolates of the human pathogen *Klebsiella pneumoniae*, causing hospital-acquired and community-acquired infections. The tested isolates were remarkable for their resistance to more than 20 commercial antibiotics of different classes. Based on previous literature data and our experiments consisting of glutamine supplementation, we suggest that both compounds release phosphinothricin—a well-known nanomolar inhibitor of glutamine synthetase—after their penetration in the bacterial cells; and, in this way, exert their antibacterial effect by negatively affecting nitrogen assimilation in this pathogen.

## 1. Introduction

An important achievement of medicine in the 20th century was the discovery of natural compounds exhibiting antimicrobial activity, and the development of new drugs based on them, which made it possible to significantly reduce mortality from infectious diseases. The widespread use of antibiotics in medical practice along with their overuse, however, led to the emergence of antibiotic-resistant pathogens and even multidrug-resistant (MDR) bugs, i.e., microorganisms that carry the genes or mutations for inactivating or making ineffective several types of antibiotics. This problem has become frightening. In 2019, more than 1.2 million people worldwide—and potentially many more—died as a direct result of the inefficacy of the antibiotics in current use to treat antibiotic-resistant bacterial infections [1]. If the spread of MDR pathogenic bacteria increases at the present rate (i.e., the rate of spread of drug resistance in bacteria continues as it is now), according to the World Health Organization (WHO) mortality from infectious diseases will attain the level of mortality caused by cardiovascular and oncological diseases by 2050 [2]. In 2009, a list of six bacteria that pose a particular threat to humans was published. The abbreviation for the bacterial species that escape the action of currently available antibiotics is known as “ESKAPE” (from *Enterococcus faecium*, *Staphylococcus aureus*, *Klebsiella pneumoniae*, *Acinetobacter baumannii*, *Pseudomonas aeruginosa*, and *Enterobacter* spp.) [3]. The list of MDR microorganisms in populations is, however, constantly updated; and, recently, the WHO published a list of twelve bacteria species for which new antibiotics are urgently needed [4].

Microorganisms with a poor response to antibiotic therapy are divided into three groups characterized by critical, high or medium threat to human health. *E. coli*, *P. aeruginosa*, *K. pneumoniae*, and *A. baumannii* are pathogens that belong to the first «critical» group, with particularly high rates of resistance to antibiotics, that the arsenal of available treatments for the diseases they cause becomes ineffective [5,6,7].

*K. pneumoniae* is a Gram-negative bacterium and among the most dangerous pathogens challenging modern medicine. It belongs to the *Enterobacteriaceae* family, is long lived, is widespread in nature, occurs on land, in aquatic environments, and is a component of the intestinal microbiota and mucosa of humans and animals [8,9]. *K. pneumoniae* is an opportunistic pathogen, causing hospital-acquired and community-acquired infections. When an infectious process occurs, the treatment is often complicated, not because of the evolution of pathogenicity factors, rather as a result of the co-existence of resistance to the beta-lactams, quinolones and aminoglycosides classes of antibiotics [10,11,12]. Therefore, *K. pneumoniae* infection usually signals a grim prognosis, with mortality up to 50% [13].

**Figure 1:**
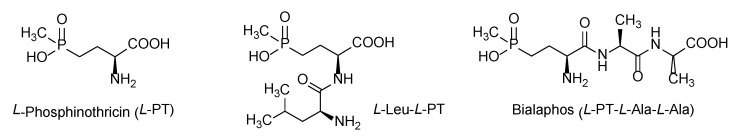
Chemical structures of *L*-phosphinothricin (*L*-PT), *L*-leucyl-*L*-phosphinothricin (*L*-Leu-*L*-PT) and *L*-phosphinothricyl-*L*-alanyl-*L*-alanine (Bialaphos, *L*-PT-*L*-Ala-*L*-Ala).

As said above, bacteria use different strategies to make antibiotics ineffective. These strategies include the biosynthesis of antibiotic-deactivating enzymes, a reduction in the intracellular antibiotic concentration, or even mutations in antibiotic targets. Among the algorithms to overcome multidrug resistance is to find new “hot spots” of bacterial metabolism and compounds that are effective on them. In this regard, in the present work, we focused on glutamine synthetase (GS) as a key target enzyme of microbial metabolism, and on the naturally occurring *L*-phosphinotricin (*L*-PT), a highly effective inhibitor of this enzyme [14,15]. The interest in glutamine synthetase as a promising target for the development of drugs towards *Mycobacterium tuberculosis* is also under investigation [16,17,18]. In this respect, virtual screening studies suggest that *L*-PT is still superior to other analogues [17]. However, in vivo studies of *L*-PT on *K. pneumoniae* MDR strains as well as on alternative approaches to heighten *L*-PT activity by improving its penetration in the bacterial cells are still lacking and lay the foundation for this study. To ensure delivery of *L*-PT (Figure 1) to *E. coli*, *K. pneumoniae* and MDR clinical isolates of *K. pneumoniae*, we used a pro-drug approach: the naturally occurring PT-containing tripeptide, Bialaphos (Figure 1), first isolated from culture filtrates of *Streptomyces viridochromogenes* [19], was therefore tested. In addition, herein, we report for the first time the synthesis and original purification of the dipeptide *L*-leucyl-*L*-PT (*L*-Leu-*L*-PT, Figure 1) and studied its antibacterial activity. Similarly, Bialaphos, once *L*-Leu-*L*-PT is taken up by the cell, is likely cleaved by intracellular peptidases, thus releasing *L*-PT [19,20], which is then responsible for inhibition of the growth of *E. coli* [14,15] and MDR *K. pneumoniae.*

## 2. Results

### 2.1. Synthesis of L-Leu-L-PT

**Scheme 1:**
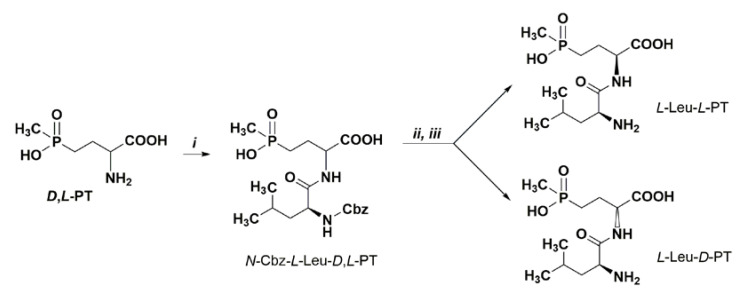
***i-*** Cbz-*L*-Leu-OSu/DME/H_2_O/Na_2_CO_3_/NaHCO_3_; ***ii-*** HBr/AcOH; ***iii-*** Dowex 50WX8 (H+), elution with H_2_O.

**Figure 2:**
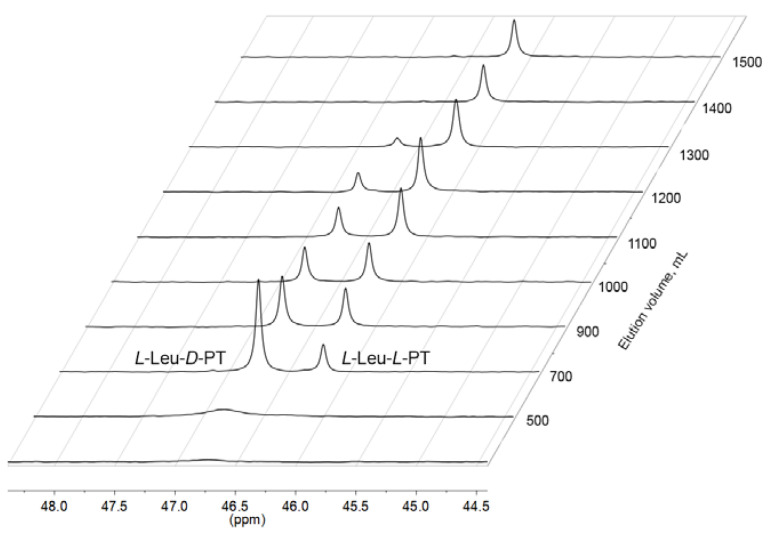
^31^P-NMR analysis of the elution profile of the separation of Leu-PT diastereomers on a Dowex-50WX8 column.

*L*-Leu-*L*-PT and *L*-Leu-*D*-PT were synthesized by the condensation of *N*-hydroxysuccinimide ester of *N*-Cbz-*L*-Leu (*N*-Cbz-*L*-Leu-OSu) with *D*,*L*-PT in water/1,2-dimethoxyethane mixture, followed by one-pot removal of Cbz-protecting group (Scheme 1). Unexpectedly, it was possible to separate the diastereomers by ion-exchange chromatography on sulfocationite Dowex-50WX8 (H+-form), by eluting the resin with a large volume of water. ^31^P-NMR analysis of the eluted fractions is depicted in Figure 2. *L*-Leu-*D*-PT eluted first, but was contaminated with some *L*-Leu-*L*-PT (approximately 10% in fraction n. 7 (700 mL, see Figure 2; for NMR spectra—Figures S6, S7 and S8). Then, a mixture of both diastereomers was eluted, and finally *L*-Leu-*L*-PT was eluted. It should be noted here that NMR chemical shifts (^1^H, ^13^C and ^31^P) of *L*,*D*- and *L*,*L*-diastereomers were different (original spectra are provided in the Supplementary information). Notably, a preliminary screening showed that only the last-eluted diastereomer (*L*-Leu-*L*-PT) possessed antibacterial activity. This is in line with the fact that only the derivatives of *L*-Glu are effective inhibitors of GS [16].

### 2.2. Bialaphos and L-Leu-L-PT Inhibit the Growth of E. coli K12 in Minimal Medium

It was shown that aminophosphonic acids penetrate poorly into bacteria [20], and *D,L*-PT is not an exception. In fact, even in minimal medium E (EG), it has poor antimicrobial activity towards *E. coli* (MIC_90_ 266–304 µg/mL, Table 1). In striking contrast, the dipeptide *L*-Leu-*L*-PT was approximately 6000-fold more active (MIC_90_ 0.04 µg/mL, Table 1), while the tripeptide Bialaphos inhibited the growth of *E. coli* in the same medium at a MIC_90_ <0.001 μg/mL (Table 1). These differences in the activity of Bialaphos and *L*-Leu-*L*-PT are likely because Bialaphos actively penetrates *E. coli* using both oligopeptide transporter *Opp* and dipeptide permease *Dpp* [21], while the dipeptide most likely uses only the latter. It should be noted that both Bialaphos and *L*-Leu-*L*-PT were poorly effective when a rich medium (Mueller–Hinton) was used. In this case, the MIC_90_ values for Bialaphos and *L*-Leu-*L*-PT were 500 and 1600 μg/mL, respectively (Table 1), which is in agreement with the known literature data disclosing poor antibacterial activity of phosphonopeptides on rich medium [20,22]. A similar relationship between the activities on minimal and rich media (Table 1) was observed in our experiments with Alaphosphin, a dipeptide containing a phosphonic moiety on the α-carbon, known to target the biosynthesis of bacteria cell wall.

### 2.3. Inhibition of the Growth of Reference Strain of Klebsiella pneumoniae ATCC 13883 with Bialaphos, L-Leu-L-PT and Antibiotics of Different Classes

**Figure 3:**
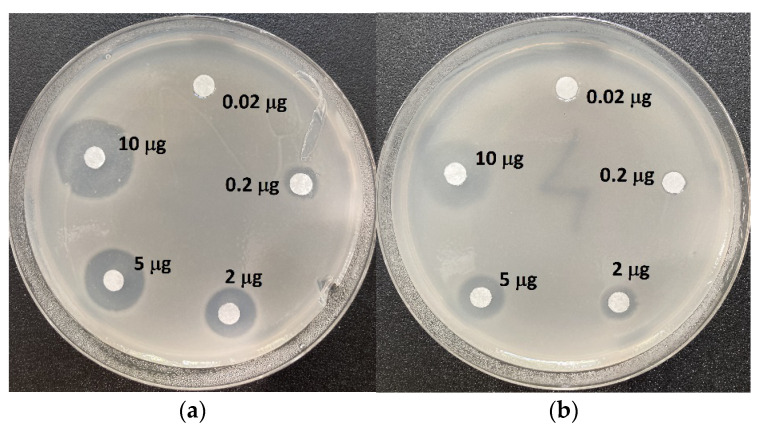
Inhibition of the growth of reference strain *Klebsiella pneumoniae* ATCC 13883 with *L*-Leu-*L*-PT and reverse of the effect with Gln. (**a**) Dose-dependent effect of *L*-Leu-*L*-PT, μg/disk; (**b**) same as (**a**), but with the agar containing 0.5 mM Gln.

As a next step, we examined the effects of Bialaphos and *L*-Leu-*L*-PT on the growth of the reference strain of *K. pneumoniae* ATCC 13883 using the agar-diffusion test. Both compounds inhibited the growth of *K. pneumoniae* in a dose-dependent manner, starting from 0.2 µg/disk, with approximately the same efficacy (Figure 3A, Figure S1A and Table 2). It should be noted that, in the case of the inhibition of *E. coli* growth, Bialaphos was at least 40-fold more active than *L*-Leu-*L*-PT (Table 1). These differences might be ascribed to the differences in the transport rate and/or the activity of peptidyl permeases in *E. coli* and *K. pneumoniae*.

The cytoplasmic cleavage of Bialaphos and *L*-Leu-*L*-PT likely releases *L*-PT (Figure S2), a very effective and specific inhibitor of GS [14,15]. Accordingly, the inhibitory activity of Bialaphos and *L*-Leu-*L*-PT was attenuated when the medium was supplemented with 0.5 mM *L*-glutamine (Figure 3B, Figure S1B). This suggests that the metabolic target of Bialaphos and *L*-Leu-*L*-PT in *K. pneumoniae* is very likely GS. Moreover, the protective effect of glutamine turned out to be dose-dependent for both peptides, i.e., Bialaphos (Figure S3) and *L*-Leu-*L*-PT (Figure S4).

**Figure 4:**
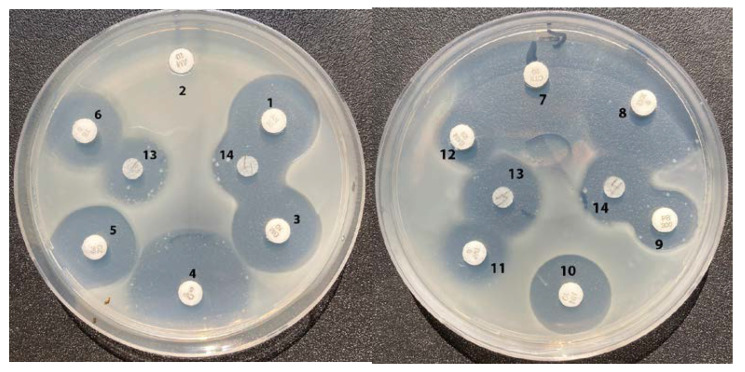
Inhibition of the growth of reference strain *Klebsiella pneumoniae* ATCC 13883 with antibiotics and peptide derivatives of phosphinothricin. (**1**) Amikacin, 30 μg/disk; (**2**) Ampicillin, 10 μg/disk; (**3**) Gentamicin, 10 μg/disk; (**4**) Ciprofloxacin, 5 μg/disk; (**5**) Cefazolin, 30 μg/disk; (**6**) Tetracycline, 30 μg/disk; (**7**) Cefotaxime, 30 μg/disk; (**8**) Levofloxacin, 5 μg/disk; (**9**) Polymyxin B, 300 IU/disk; (**10**) Tobramycin, 10 μg/disk; (**11**) Trimethoprim, 5 μg/disk; (**12**) Ampicillin/Sulbactam, 10/10 μg/disk; (**13**) Bialaphos, 5 μg/disk; (**14**) *L*-Leu-*L*-PT, 5 μg/disk.

The growth of the reference strain of *K. pneumoniae* ATCC 13883 is inhibited by many antibiotics, such as aminoglycosides, cephalosporins, fluoroquinolones, and tetracyclines. Amongst the antibiotics used in this work, the most effective were Ciprofloxacin, Cefotaxime, and Levofloxacin (Figure 4—disks 4, 7, 8, respectively; and Table S1), while the activity of the other tested antibiotics was approximately the same as that of Bialaphos and *L*-Leu-*L*-PT (Figure 4, Table S1). It is worth point out that Ampicillin (Figure 4, disk 2 and Table S1) was active only when used in combination with Sulbactam, the β-lactamase inhibitor (Figure 4, disk 12 and Table S1) and even in this case it exerted a limited effect as it can be deduced from the small inhibition halo. All the antibiotics on disks were applied using the doses typically tested in antibiotics susceptibility testing, ranging from 5 to 30 µg/disk [23]. Bialaphos and *L*-Leu-*L*-PT were applied to disks at the lowest concentration in this range (5 μg/disk), while the zones of inhibition were distinct and ranged from 18 to 23 mm in diameter. When analyzed in vitro, on average, the dose tested is more closely resembling the concentration attained when these antibiotics are administrated in patients as medicines.

### 2.4. Growth Inhibition of Multidrug-Resistant Klebsiella pneumoniae Clinical Isolates

MDR bacteria are threatening, especially when isolated in health care settings (hospitals, nursing homes, etc.), amongst immunocompromised patients. Carbapenems-resistant strains of *K. pneumoniae* are classified by the WHO as the first and most dangerous group of drug-resistant bacteria [4] since carbapenems are the last resort of β-lactam antibiotics to treat MDR infections.

Here, we tested Bialaphos and *L*-Leu-*L*-PT on three MDR strains that are clinical isolates of *K. pneumoniae* (Table S2, strains 1161, 1158 and 1133) from the Shumakov Centre of Transplantology (Moscow, Russia). First, we showed that Bialaphos and *L*-Leu-*L*-PT inhibited the growth of all three MDR strains in a dose-dependent manner at 0.5–10 μg/disk (Table 2, Figure S5). It is worth noting that under the assay conditions used in this study *L*-Leu-*L*-PT is slightly more potent than Bialaphos in the clinical isolates (Table 2, Figure S5). At present, we cannot provide an explanation for this, but cannot exclude that this difference may be due to mutations that affect the transport rate of dipeptide and oligopeptide transporters in the MDR strains respect to the reference strain ATCC 13883.

**Figure 5:**
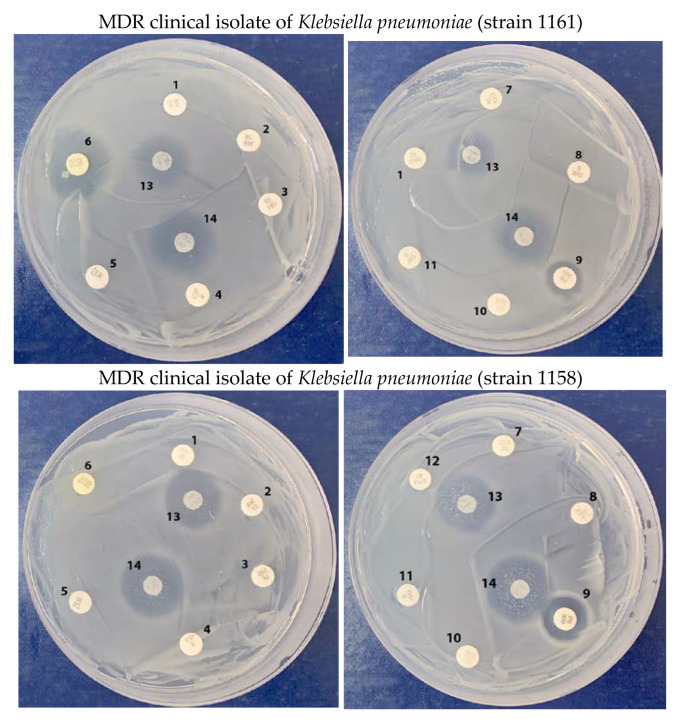

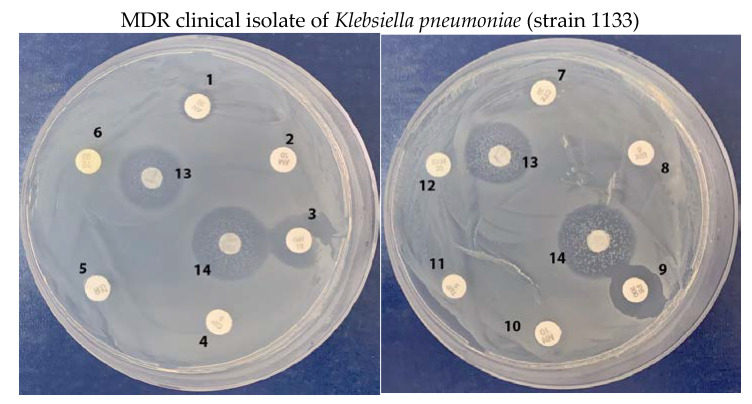
Inhibition of the growth of clinical isolates of MDR K. pneumoniae with antibiotics and peptide derivatives of phosphinothricin. (**1**) Amikacin, 30 μg/disk; (**2**) Ampicillin, 10 μg/disk; (**3**) Gentamicin, 10 μg/disk; (**4**) Ciprofloxacin, 5 μg/disk; (**5**) Cefazolin, 30 μg/disk; (**6**) Tetracycline, 30 μg/disk; (**7**) Cefotaxime, 30 μg/disk; (**8**) Levofloxacin, 5 μg/disk; (**9**) Polymyxin B, 300 IU/disk; (**10**) Tobramycin, 10 μg/disk; (**11**) Trimethoprim, 5 μg/disk; (**12**) Ampicillin/Sulbactam, 10/10 μg/disk; (**13**) Bialaphos, 5 μg/disk; (**14**) L-Leu-L-PT, 5 μg/disk.

Finally, we compared the inhibitory activity of Bialaphos and *L*-Leu-*L*-PT against the MDR strains 1161, 1158 and 1133 of *K. pneumoniae* with that of twelve commercially available antibiotics belonging to different classes, i.e., aminoglycosides, β-lactams, cephalosporins, fluoroquinolones, tetracyclines, etc. In these experiments, 5.0 μg/disk of Bialaphos and *L*-Leu-*L*-PT were used. Notably, this amount is much lower than the amount needed to test most of the other antibiotics. In agreement with the previous results (Table 2, Figure S5), Bialaphos and *L*-Leu-*L*-PT were quite potent on all the three MDR strains (Figure 5, Table S1) whereas, among the tested antibiotics, only Polymyxin B inhibited the growth in all these strains. The growth of strain 1161 was also inhibited by Tetracycline, while that of strain 1133 was inhibited by Gentamicin (Figure 5, Table S1). Although the molecular mechanisms responsible for the multidrug resistance in these three strains of *K. pneumoniae* has not yet been analyzed in detail, Bialaphos and *L*-Leu-*L*-PT turned out to be effective inhibitors of the growth of all these MDR strains. According to the current knowledge [16,17,18], based on studies carried out in other microorganisms using Bialaphos, the inhibition of the growth is a consequence of GS targeting, thus eventually affecting Gln metabolism and nitrogen assimilation [24].

## 3. Discussion

After the discovery of the penicillin by Fleming, microorganisms attracted researchers’ interest as the most important source of biologically active compounds of different classes. Many of these substances are used as antibiotics per se, acting on the biosynthesis of the bacterial cell wall, protein biosynthesis, bacterial DNA replication, etc.; or serve as lead compounds for the targeted synthesis of new compounds with stronger antibacterial activity at lower doses.

**Figure 6:**
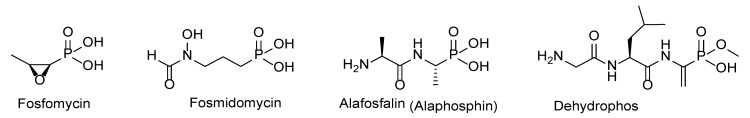
Chemical structures of Fosfomycin, Fosmidomycin, Alaphosphin (Alafosfalin) and Dehydrophos.

Microorganisms also produce a wide range of substances with an unusual phosphorus-carbon bond (*P*-*C* bond), which is biochemically highly stable. Antibacterial compounds with *P*-*C* bond have found practical application in medicine and agriculture, since the phosphorus-containing group can mimic a phosphate monoester or a tetrahedral intermediate (or reaction transition state) of the carboxyl group transformations [25,26]. Notable examples (Figure 6) include the antibiotic Fosfomycin (an irreversible inhibitor of muramyl ligase A [27], the first enzyme of peptidoglycan synthesis), which is used for the treatment of cystitis; the antimalarial Fosmidomycin (an inhibitor of 1-deoxy-*d*-xylulose-5-phosphate reductoisomerase [28,29], an essential enzyme of the non-mevalonate pathway of isoprenoid biosynthesis), which is also active against different enterobacteria, but not against Gram-positive microorganisms or anaerobes; the already mentioned Alaphosphin (Section 2.2); and the commercial herbicide Bialaphos (Figure 1), a naturally occurring tripeptide, which, upon cleavage in the cell, gives rise to *L*-PT (Figure 1)—a very efficient inhibitor of glutamine synthetase [14,15].

Microorganism genome mining is the modern approach to discover phosphorus-containing antibacterials. It is based on the exploitation of the genomic information to gain knowledge on the biosynthesis of compounds with *P*-*C* bond. Most of the known phosphonates are derived from phosphoenolpyruvate by isomerization to phosphonopyruvate (universal building block used for the biosynthesis of compounds with *P*-*C*-bond), which is catalyzed by the enzyme phosphoenolpyruvate mutase, followed by subsequent enzymatic decarboxylation, catalyzed by phosphonopyruvate decarboxylase, which gives rise to phosphonoacetaldehyde [25,26,30]. Phosphonoacetaldehyde can then undergo a large spectrum of transformations serving as another universal biochemical building block. The analysis in genome databases of the genes coding for these two enzymes, as well as of their homologues, enabled to conclude that up to 10–15% of bacterial species can produce phosphonates. Therefore, the genome mining of 10,000 actinomycetes led to rediscovering of many old phosphonates, as well as 19 new compounds, including those with antibacterial activity [31].

The enzymes involved in the biosynthesis of peptidoglycan (murein), an important constituent of the bacterial cell wall, are common targets of different amino acid-derived compounds with antibacterial activity [32]. Aminophosphonic acids, with a phosphorus-containing group replacing the carboxyl-one (-COOH) are amongst these amino acid analogues and derivatives. However, aminophosphonates, as such, poorly penetrate in the cells of eukaryotes and prokaryotes; thereby to deliver these compounds some modifications of the molecule are required. This could explain why many of the naturally produced aminophosphonates are found in nature as short peptides, which are taken up by bacteria and fungi via peptidyl permeases [33]. Subsequent intracellular cleavage of these penetrated phosphonate-containing peptides by cellular peptidases releases the active compound, the aminophosphonic acid. Among the first examples of such a «Troyan-horse» (prodrug) strategy for the design of the antibacterial agent was the synthesis of Alaphosphin (Figure 6). Following intracellular cleavage, Alaphosphin releases the phosphonic analogue of alanine, a highly effective inhibitor of alanine racemase, thus leading to the inhibition of the biosynthesis of the bacterial cell wall and, consequently, the inhibition of bacteria growth [22]. Dehydrophos (Figure 6) may be considered as a “double prodrug” because it penetrates the bacterial cell using peptidyl permeases and, after the cleavage of the peptide bond, provides the phosphonic analogue of dehydroalanine. This analogue undergoes spontaneous rearrangement into methyl acetylphosphonate [34], which is an analogue of pyruvic acid and a very strong inhibitor of pyruvate dehydrogenase [35] and ref. within.

Herein, we used *L*-PT (Figure 1) as a phosphorus-containing antibacterial to inhibit the growth of the reference *E. coli* K12 strain MG1655, the reference strain of *K. pneumoniae* ATCC 13883 as well as three clinical MDR strains of *K. pneumoniae* available in the collection of the Shumakov Federal Research Centre of Transplantology and Artificial Organs. *L*-PT is known to irreversibly inhibit GS, a key intracellular enzyme required for the synthesis of glutamine from glutamate and ammonia, and in that respect, it is fundamental in microbial nitrogen assimilation. The inhibition mechanism has been elegantly elucidated by demonstrating that the *C-P* group mimics the phosphorylated intermediate of glutamate formed during the enzymatic reaction, and that this intermediate does not allow the completion of the enzymatic reaction [14,15]. To deliver the poorly penetrating *L*-PT in *E. coli* and *K. pneumoniae,* we incorporated it into a synthetic dipeptide, i.e., *L*-Leu-*L*-PT, as well as used it in the form of the commercially available herbicide Bialaphos (Figure 1), which is a naturally occurring *L*-PT-containing tripeptide with a well-characterized biosynthesis [36] and low toxicity to vertebrates. In fact, the acute oral LD_50_ values of Bialaphos for male and female rats are 268 and 404 mg/kg, respectively; and the acute oral LD_50_ value for chicken is greater than 5000 mg/kg. Bialaphos was also shown not to be mutagenic in the Ames assay [37]. Furthermore, Phosalacine, a tripeptide consisting of *L*-PT-*L*-Ala-*L*-Leu, was also shown to lack toxicity in mice when administered at 500 mg/kg [38].

Once taken up by bacteria, both Bialaphos and *L*-Leu-*L*-PT are proposed to be cleaved by peptidases thus giving rise to *L*-PT (Figure S2). To the best of our knowledge, the dipeptide *L*-Leu-*L*-PT does not exist in nature. Therefore, herein, we show for the first time its synthesis and purification and tested its antimicrobial potential on the *E. coli* K12 reference strain MG1655. Furthermore, we observed that *L*-Leu-*L*-PT and Bialaphos were also effective at inhibiting the growth of the reference strain *K. pneumoniae* ATTC 13,883 and, more importantly, the growth of MDR *K. pneumoniae* isolates. We observed that a few μg/disk of Bialaphos and *L*-Leu-*L*-PT inhibit the growth of three clinical isolates of *K. pneumoniae* (MDR strains 1161, 1158 and 1133), otherwise resistant to more than twenty commercially available antibiotics of different classes, including carbapenems Imipenem and Meropenem (Table S2). This is a finding that we regard as remarkable.

## 4. Materials and Methods

### 4.1. Materials

Sodium salts of Bialaphos and *D,L*-phosphinothricin (Glufosinate-ammonium) were obtained from Santa Cruz Biotechnology; Alaphosphin was from Fluka. Agar agar powder No 1 for bacteriology was from LobaChemie. *N*-(Benzyloxycarbonyl)-*L*-leucine *N*-hydroxysuccinimide ester (Z-*L*-Leu-OSu) was synthetized according to [39] and was freshly recrystallized from *i*-PrOH before use. All other reagents, salts and solvents were of highest purity and used as supplied by Aldrich and Acros.

TLC was carried out on Cellulose F (Merck, Germany) in *i*-PrOH–25% NH_4_OH–H_2_O = 7:1:2. *L*-Leu-*L*-PT was detected on TLC plates following staining with ninhydrin (0.4% in acetone).

Ion-exchange chromatography was carried out on Dowex 50WX8, H+-form, 100–200 mesh (BioRad) using water for elution.

NMR spectra were recorded on a Bruker AM-300 (300.13 MHz for ^1^H, 75.43 MHz for ^13^C, and 121.44 MHz for ^31^P) using D_2_O as a solvent with sodium 3-trimethyl-1-propanesulfonate (DSS) as internal standard, or 85% H_3_PO_4_ as external standard. Chemical shifts are given in parts per million (ppm), the letter “*J*” indicates spin-spin coupling constants which are given in Hertz (Hz).

### 4.2. Synthesis of L-Leucyl-L-Phosphinothricin

A solution of glufosinate-ammonium (400 mg, 2.0 mmol) in 0.5 M NaOH (8 mL) was concentrated *in vacuo*, the residue was co-evaporated in vacuo with water (2 × 10 mL) and to the residue water (6.5 mL), NaHCO_3_ (84 mg, 1.0 mmol), Na_2_CO_3_ (106 mg, 1.0 mmol) and 1,2-dimethoxyethane (1.0 mL) were added. To the obtained solution, *N*-Cbz-*L*-Leu-OSu (780 mg, 2.15 mmol) in 1,2-dimethoxyethane (4.0 mL) was added and the resulting solution was stirred overnight at 20 °C. The reaction mixture was concentrated in vacuo, the residue was dissolved in water (15.0 mL), acidified with 37% HCl (1.5 mL) and the separated oil was extracted with EtOAc (4 × 8.0 mL). The combined EtOAc extract were washed with water (3.0 mL), brine (2 × 7.0 mL) and dried (MgSO_4_). The solvent was removed in vacuo and the residue was dried in vacuo (1.0 Torr) at 40 °C for 1 h. The resulting foam was dissolved in glacial AcOH (2.5 mL), then anisole (8 drops) and 35% HBr/AcOH (2.0 mL) were added; the reaction mixture was incubated at 20 °C for 2.5 h (i.e., until the evolution of CO_2_ ended), pooled into abs. Et_2_O (60 mL) and left overnight at −20 °C. Solvents were decantated, the residual oil was co-evaporated in vacuo with water (2 × 10 mL), the residue was dissolved in water (10 mL) and applied on a Dowex 50WX8 column (V = 8.0 mL). Column was eluted with water (2.5 L) and each fraction (100 mL) was concentrated in vacuo *to* 10 mL and then ninhydrin-positive fractions (from 5 to 25) were analyzed by ^31^P-NMR (Figure 2). Fraction 5 contained pure *L*-Leu-*D*-PT (25 mg, yield 4.3%). Fractions from 6 to 14 contained a mixture of both diastereomers (358 mg, yield 60.9%). The NMR spectra of fractions 7 and 11 are depicted in the Supplement (Figures S6, S7, S8, S9 and S10). Fractions from 15 to 25 contained pure *L*-Leu-*L*-PT (125 mg, yield 21.3%): *R_f_* 0.49. ^1^H NMR (300.13 MHz, D_2_O): δ = 4.42 (1H, dd, ^3^*J*_HH_ = 8.1 Hz, ^3^*J*_HH_ = 5.1 Hz, CHCOOH); 4.06 (1H, dd, ^3^*J*_HH_ = 7.7 Hz, ^3^*J*_HH_ = 6.7 Hz, CHC(O)NH); 2.17–1.88 (2H, m, CH_2_CH_2_P), 1.83–1.58 (5H, m, (CH_3_)_2_CHCH_2_ + CH_2_P); 1.30 (3H, d, ^2^*J*_HP_ = 13.6 Hz, CH_3_P); 0.96 (6H, t, ^3^*J*_HH_ = 6.1 Hz, (CH_3_)_2_CH-). ^13^C NMR (75.43 MHz, D_2_O): δ = 175.86; 170.97; 54.83 (d, ^3^*J*_PC_ = 15.8 Hz); 52.55; 40.49; 27.70 (d, ^1^*J*_PC_ = 92.0 Hz); 25.04; 24.42; 22.31, 21.80; 15.32 (d, ^1^*J*_PC_ = 92.4 Hz). ^31^P NMR (121.44 MHz, D_2_O): δ = 46.16. For original NMR spectra of *L*-Leu-*L*-PT, see Figures S8, S11 and S12. HRMS (ESI-MS): found *m/z* 295.1417; calc. for C_11_H_23_N_2_O_5_P [M+H]^+^ 295.1423.

### 4.3. The Microdilution Method to Determine the Antimicrobial Activity of Tested Compounds against Escherichia coli

The minimum inhibitory concentration of 90% colony-forming units (MIC_90_) of the test strain *E. coli* K12 MG1655 was determined by the broth microdilution method in the minimal medium EG containing MgSO4•7H_2_O (0.2 g), citric acid•H_2_O (2.0 g), anhydrous K_2_HPO_4_ (10.0 g), NaNH_4_HPO_4_•H_2_O (3.5 g), and glucose (4.0 g), milliQ water (1.0 L) final pH 7, prepared as described earlier [40]. Briefly, overnight cultures (2 mL) of *E. coli* K12 strain MG1655 were centrifuged at 3500 rpm for 15 min at 15 °C and the cell pellets resuspended in an equivalent volume of physiological solution (9 g/L NaCl). The OD_600_ was then brought to 1.0. The resuspended cells were inoculated (1:25) in minimal medium EG (2 mL) and grown at 37 °C up to OD_600_ = 0.5, then diluted (1:25) in the same minimal medium and dispensed in a 96-well microplate previously set up with the appropriate serial dilutions of the compounds to be tested (*L*-Leu-*L*-PT, Bialaphos, *D,L*-PT, Alaphosphin). The number of colony-forming units (CFU)/mL at time zero was between 0.5–1.0 × 10^6^/well and the final volume in each well was 200 µL. The microplate was incubated at 37 °C for 24 h in the microplate reader Varioskan Lux (Thermo Scientific) and every hour the OD_600_ was automatically recorded. MIC_90_ was calculated at 22 h from the time of the inoculum using the equation: % inhibition = [1 − (OD_600_treated/OD_600_untreated)] × 100.

### 4.4. The Agar Diffusion Method to Analyze Antimicrobial Activity of Tested Compounds against Klebsiella pneumoniae

The reference strain *K. pneumoniae* ATCC 13883 and MDR clinical isolates from patients of Shumakov Federal Research Centre of Transplantology and Artificial Organs (Moscow, Russia) were used. Species identification of clinical isolates of *K. pneumoniae* and their sensitivity to antibiotics were determined on an automatic bacteriological analyzer for the identification of microorganisms (MicroScan WalkAway-96 plus System, Beckman Coulter, USA) following the manufacturer’s instructions.

Bialaphos, *L*-Leu-*L*-PT and twelve commercial antibiotics were tested as follows. Different amounts of the substances under testing were applied to paper discs, the discs were air dried and placed on the surface of an agar plate containing M9 medium with the following composition: Na_2_HPO_4_•7H_2_O (12.8 g), anhydrous K_2_HPO_4_ (3.0 g), NaCl (0.5 g), NH_4_Cl (1.0 g), MgSO4•7H_2_O (0.5 g), CaCl_2_ (15 mg), glucose (4.0 g), thiamine (1 mg), agar (15 g) brought to 1 L with milliQ water and to a final pH 7.2. The plates were previously seeded with a lawn of *K. pneumoniae* ATCC 13883 or one of the clinical isolates; the seeding density was 10^6^ bacteria per cm^2^ of the agar surface. The plates were incubated for 20 h at 37 °C. Already prepared disks with the other antibiotics were from Becton, Dickinson & Co., Franklin Lakes, NJ, USA. The antibiotic activity of all the compounds was tested by the agar diffusion method [41] and determined based on the presence and size of non-growth zones around the disks.

## 5. Conclusions

Phosphinothricin (PT), an inhibitor of glutamine synthetase (GS), in the form of its actively penetrating di- and tripeptides, i.e., *L*-Leu-*L*-PT and Bialaphos, is effective in inhibiting multidrug-resistant (MDR) clinical isolates of *K. pneumoniae*, otherwise not sensitive to twenty four commonly used antibiotics of different classes. Our data suggest that nitrogen assimilation via glutamine biosynthesis is of crucial importance for *K. pneumoniae* and might be considered as a target to affect the growth of MDR strains of this pathogen. Furthermore, we describe the original synthesis and purification of *L*-Leu-*L*-PT.

## Figures and Tables

**Table 1:** Minimum inhibitory concentration (MIC_90_, µg/mL) of the compounds tested on *E. coli* K12 MG1655 determined by the broth microdilution method in minimal (EG) and rich (Mueller–Hinton) media.

Compound		Medium	
		Minimal EG	Mueller–Hinton
Amino acid	*D,L*-Phosphinothricin (*D,L*-PT)	266–304	n.d.
Dipeptides	*L*-Ala-*L*-aminoethylphosphonic acid (Alaphosphin)	0.001–0.01 *	1000 (MIC_50_)
	*L*-Leu-*L-*PT	0.04	1600
Tripeptide	Bialaphos (*L*-PT-*L*-Ala-*L*-Ala)	<0.001	500 (MIC_87_)

* Alaphosphin has a MIC_99.9_ of 0.25 µg/mL on *E. coli* NCTC 10,418 by agar incorporation method on minimal medium [22].

**Table 2:** Dose-dependent inhibition of the growth of clinical isolates of *K. pneumoniae* MDR strains 1161, 1158 and 1133 with Bialaphos and L-Leu-L-PT (yellow background); reference strain ATCC 13883 with Bialaphos (blue background) and L-Leu-L-PT (green background). The displayed values from two dishes.

PT Derivatives (μg/Disk)	Strain 1161	Strain 1158	Strain 1133	ATCC 13883
	Diameter of Inhibition Zones, mm			
Bialaphos, 0.01	0/0	0/0	0/0	0/0
Bialaphos, 0.1	0/0	0/0	0/0	9/8 **
Bialaphos, 0.5	0/0	10/9	13/15	n.t.
Bialaphos, 1.0	0/0	8/7	13/13	15/14 ***
Bialaphos, 5.0	14/13	18/15	16/17	20/20
Bialaphos, 10.0	18/17	21/19	21/21	25/27
*L*-Leu-*L*-PT, 0.01	0/0	0/0	0/0	0/0
*L*-Leu-*L*-PT, 0.1	0/0	0/0	11/12	8/7 **
*L*-Leu-*L*-PT, 0.5	10/11	8/9	12/14	n.t.
*L*-Leu-*L*-PT, 1.0	13/13	13/13	16/16	12/10 ***
*L*-Leu-*L*-PT, 5.0	16/20	20/20	20/22	14/12
*L*-Leu-*L*-PT, 10.0	18/21	23/23	25/25	19/20

Blue background of Table is illustrated by Figure 2; green—by Figure S1; yellow—by Figure S5. In these figures, only one of the two dishes is shown. **—0.2 μg/disk; ***—2.0 μg/disk; n.t.—not tested.

## Data Availability

The data presented in this study are available in supplementary material.

## Supplementary Materials

The following supporting information can be downloaded at: https://www.mdpi.com/article/10.3390/molecules28031234/s1, Figure S1: Inhibition of the growth of reference strain *Klebsiella pneumoniae* ATCC 13883 with Bialaphos and the reverse of the effect with Gln.; Figure S2: Proposed mechanism of antibacterial activity of Bialaphos and *L*-Leu-*L*-PT.; Figure S3: Glutamine reverses Bialaphos-induced inhibition of the growth of reference strain of *Klebsiella pneumoniae* ATCC 13883 doze-dependently.; Figure S4: Glutamine reverses *L*-Leu-*L*-PT-induced inhibition of the growth of reference strain of *Klebsiella pneumoniae* ATCC 13883 doze-dependently.; Figure S5: Doze-dependent inhibition of the growth of multi-drug resistant clinical isolates of *Klebsiella pneumoniae* with Bialaphos and *L*-Leu-*L*-PT.; Figure S6: ^1^H-NMR spectrum of *L*-Leu-*D*-PT with about 10% of *L*,*L*-diastereomer (fraction n. 7).; Figure S7: ^13^C-NMR spectrum of *L*-Leu-*D*-PT with about 10% of *L,L*-diastereomer (fraction n. 7).; Figure S8: Combined ^31^P-NMR spectra of *L*-Leu-*L*-PT, *L*-Leu-*D*-PT with about 10% of *L,L*-diastereomer (fraction n. 7) and *L*-Leu-*rac*-PT (fraction 11).; Figure S9: ^1^H-NMR spectrum of *L*-Leu-*rac*-PT (fraction n. 11).; Figure S10: ^13^C-NMR spectrum of *L*-Leu-*rac*-PT (fraction n. 11).; Figure S11: ^1^H-NMR spectrum of *L*-Leu-*L*-PT.; Figure S12: ^13^C-NMR spectrum of *L*-Leu-*L*-PT.; Table S1: Inhibition of the growth of clinical isolates of multi-drug resistant *Klebsiella pneumoniae* and reference strain ATCC 13883 with Bialaphos, *L*-Leu-*L*-PT and antibiotics of different classes.; Table S2: Multi-drug resistant clinical isolates of *Klebsiella pneumoniae*. R–resistant; S–sensitive; Antibiotics verified in this study are in the left column and are in blue.
