# Supplementary material for: Antibacterial Activity of Peptide Derivatives of Phosphinothricin against Multidrug-Resistant Klebsiella pneumoniae
Source: Molecules. 2023 Jan 27;28(3):1234. doi: 10.3390/molecules28031234 (PMC9920448; doi:10.3390/molecules28031234)
Supplement: Supplementary file 1 [file molecules-28-01234-s001.zip › molecules-2127580-supplementary.pdf]

Supplementary materials

Antibacterial Activity of Peptide Derivatives of Phosphinothricin against Multidrug-Resistant *Klebsiella pneumoniae*

Marija V. Demiankova [1,†], Fabio Giovannercole [2,†,‡], Maxim A. Khomutov [3], Arthur I. Salikhov [3], Laura Onillon [2,§], Vladimir T. Valuev-Elliston [3], Byazilya F. Vasilieva [1], Elena N. Khurs [3], Nina I. Gabrielyan [4], Sergey N. Kochetkov [3], Olga V. Efremenkova [1,*], Daniela De Biase [2,*] and Alex R. Khomutov [3,*]

[1] Gause Institute of New Antibiotics, Bol'shaya Pirogovskaya 11, 119021 Moscow, Russia; mary_bunny@mail.ru (M.V.D.); bfvas@yandex.ru (B.F.V.)

[2] Department of Medico-Surgical Sciences and Biotechnologies, Sapienza University of Rome, Corso della Repubblica 79, I-04100 Latina, Italy; fabio.giovannercole@gmail.com (F.G.); laura.onillon@ifremer.fr (L.O.)

[3] Engelhardt Institute of Molecular Biology, Russian Academy of Sciences, Vavilov Street 32, 119991 Moscow, Russia; makhomutov@mail.ru (M.A.K.); asalihov93@gmail.com (A.I.S.); gansfaust@mail.ru (V.T.V.-E.); enkhurs@yandex.ru (E.N.K.); kochet@eimb.ru (S.N.K.)

[4] Academician V.I. Shumakov Federal Research Centre of Transplantology and Artificial Organs,
Moscow 123182, Russia; labgso@mail.ru

* Correspondence: ovefr@yandex.ru (O.V.E.); daniela.debiase@uniroma1.it (D.D.B.); alexkhom@eimb.ru (A.R.K.)

† These authors contributed equally to this work.

‡ Present address: Département de Biologie, Université de Namur, Rue de Bruxelles 61, 5000 Namur, Belgium

§ Present address: IHPE UMR 5244, Université de Montpellier, Place Eugène Bataillon CC 80, F-34095 Montpellier CEDEX 5, France

Keywords: multidrug-resistant bacteria; *Klebsiella pneumoniae*; *Escherichia coli*; Glutamine synthetase; phosphinothricin; Bialaphos

---

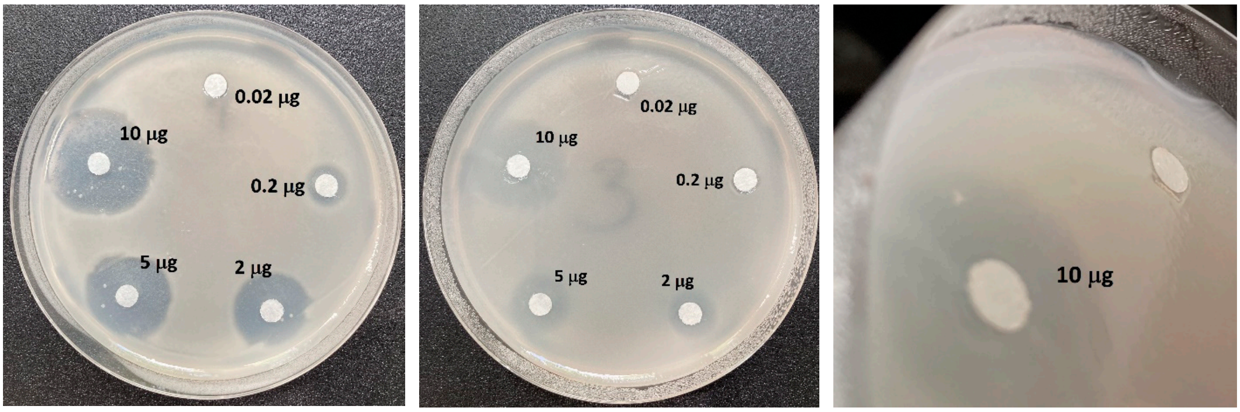

(a) (b) (c)

Figure S1. Inhibition of the growth of reference strain *Klebsiella pneumoniae* ATCC 13883 with Bialaphos and the reverse of the effect with Gln. **(a)** – dose-dependent effect of Bialaphos, μg per disk; **(b) –** same as (a), but with the agar containing 0.5 mM Gln; **(c)** – zoomed (b) taken with angle.

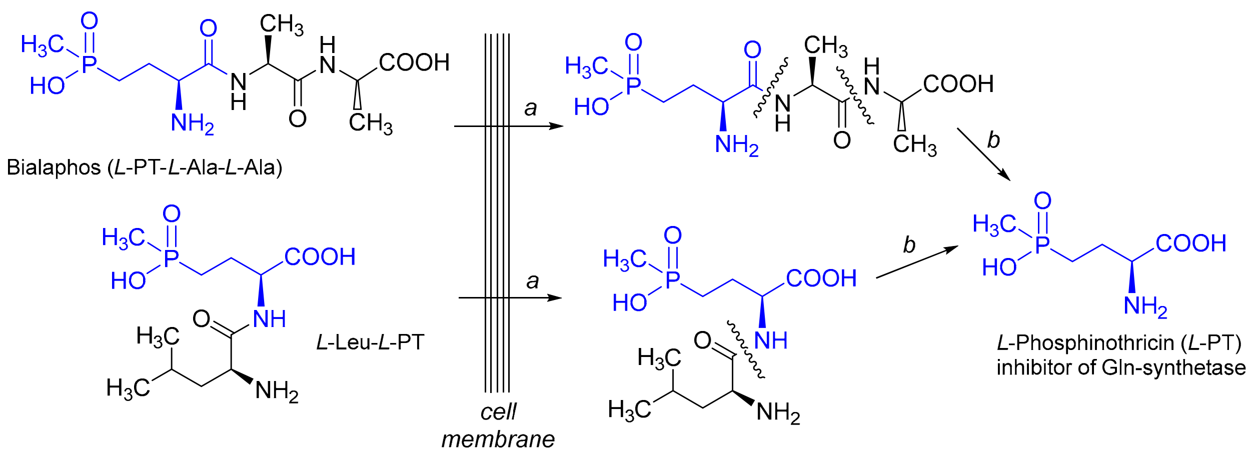

Figure S2. Proposed mechanism of antibacterial activity of Bialaphos and *L*-Leu-*L*-PT. **a–** peptidyl permeases; **b–** peptidases.

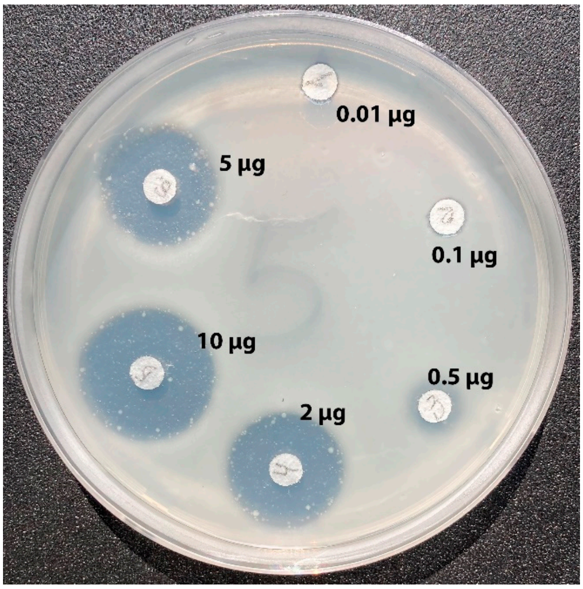

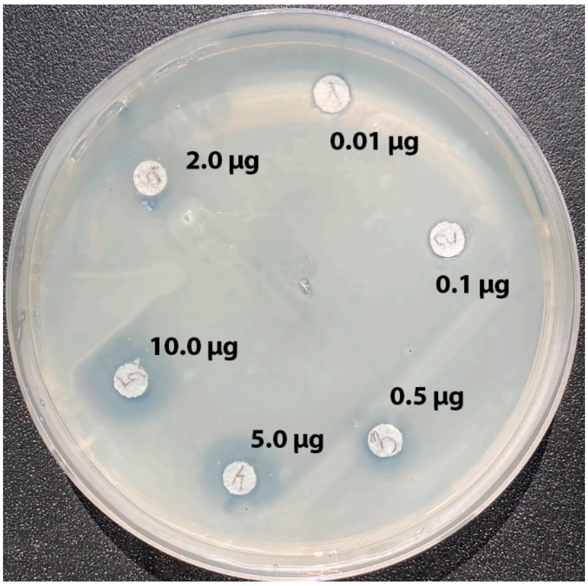

(a) **(b)**

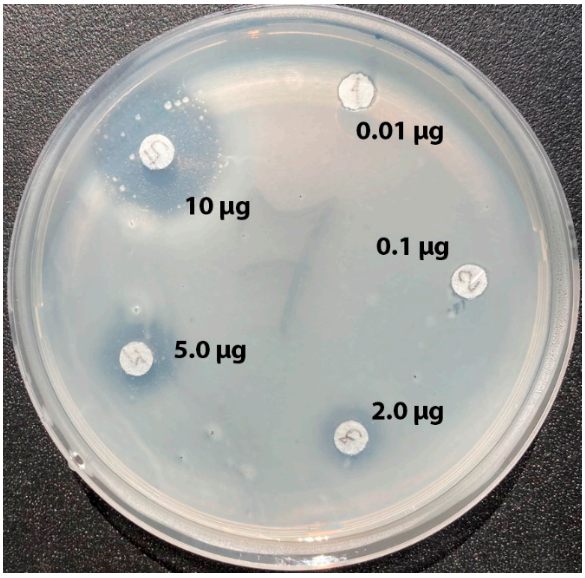

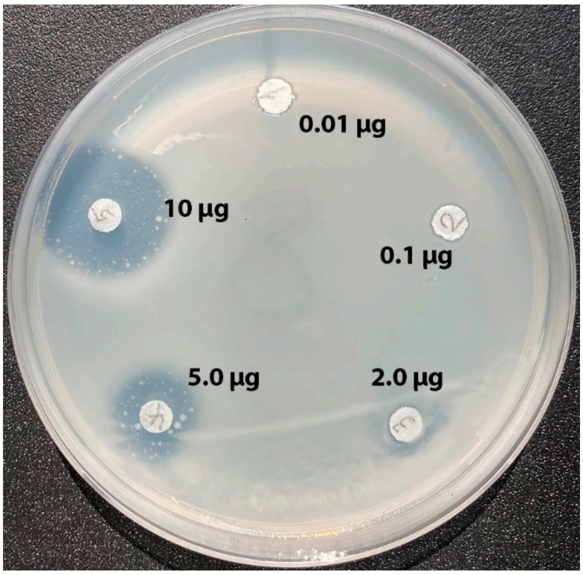

(c) **(d)**

Figure S3. Glutamine reverses Bialaphos-induced inhibition of the growth of reference strain of *Klebsiella pneumoniae* ATCC 13883 doze-dependently. Figures at dishes show the amount of bialaphos applied on the disk. **(A) –** no Gln in agar; **(B) –** agar containing 0.5 mM Gln; **(C)** – agar containing 0.1 mM Gln; **(D)** – agar containing 0.02 mM Gln.

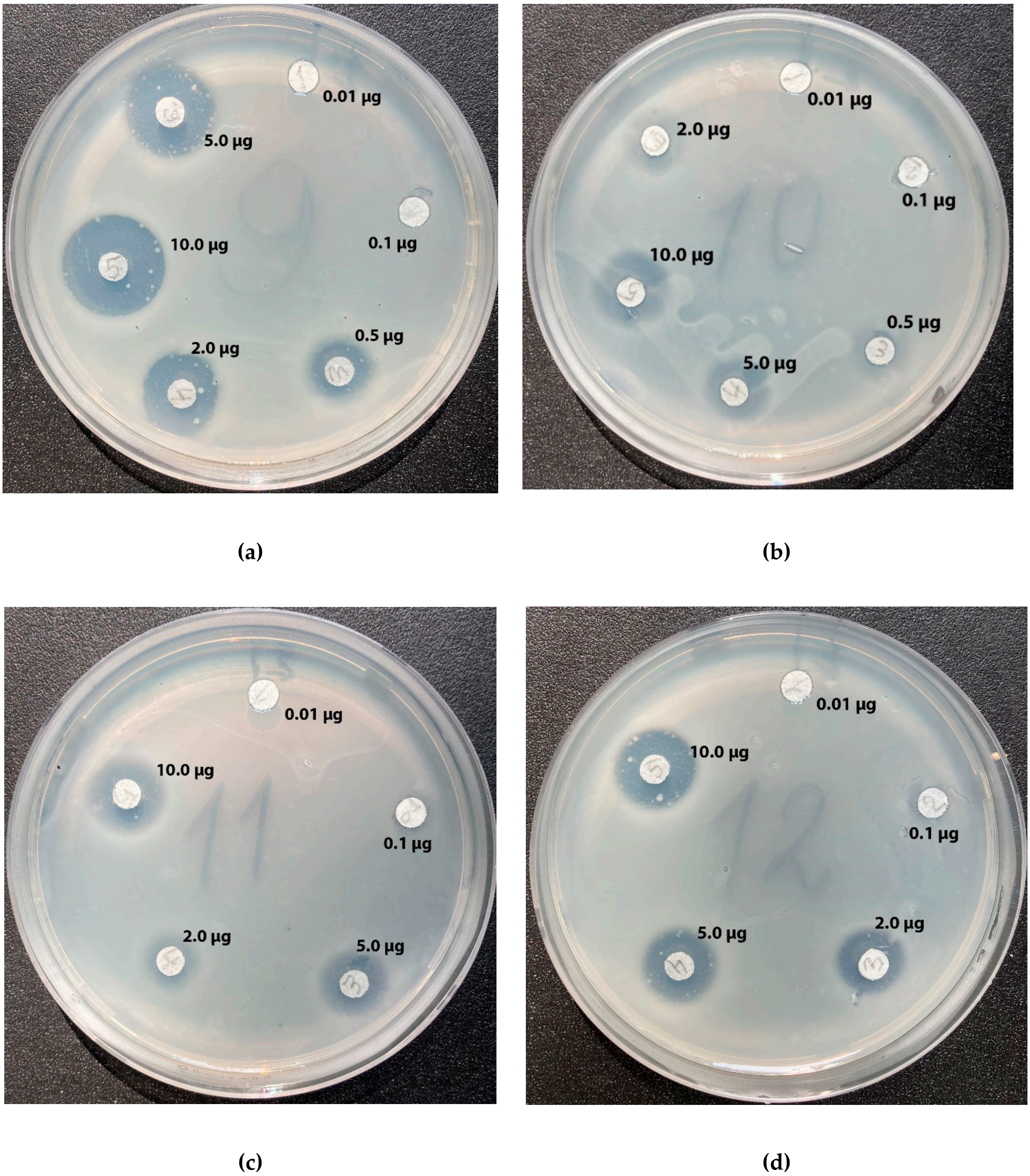

Figure S4. Glutamine reverses *L*-Leu-*L*-PT-induced inhibition of the growth of reference strain of *Klebsiella pneumoniae* ATCC 13883 doze-dependently. Figures at dishes show the amount of *L*-Leu-*L*-PT applied on the disk. **(A) –** no Gln in agar; **(B) –** agar containing 0.5 mM Gln; **(C)** – agar containing 0.1 mM Gln; **(D)** – agar containing 0.02 mM Gln.

MDR clinical isolate of *Klebsiella pneumoniae* (strain 1161)

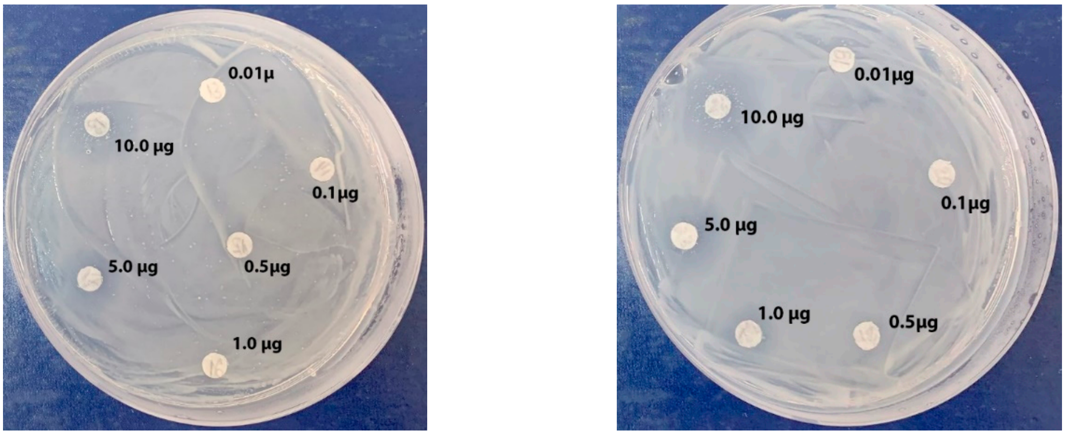

MDR clinical isolate of *Klebsiella pneumoniae* (strain 1158)

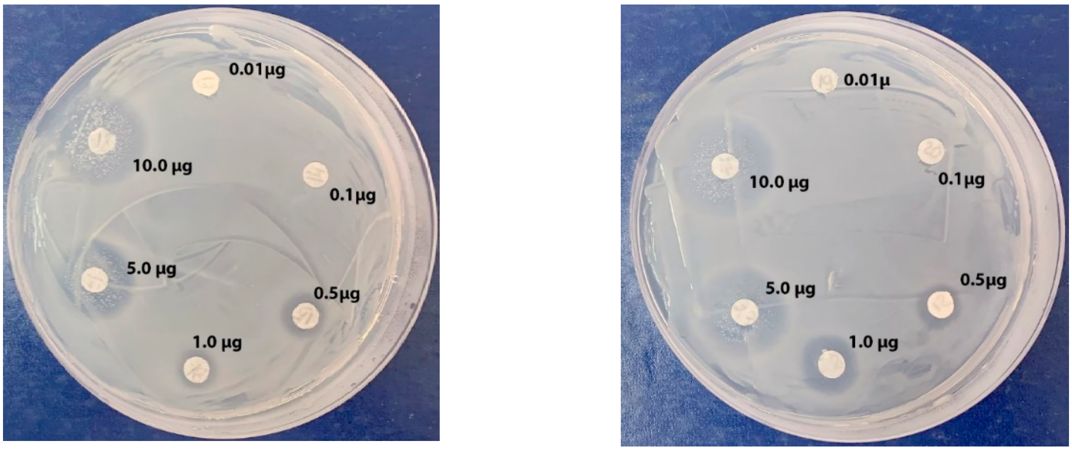

MDR clinical isolate of *Klebsiella pneumoniae* (strain 1133)

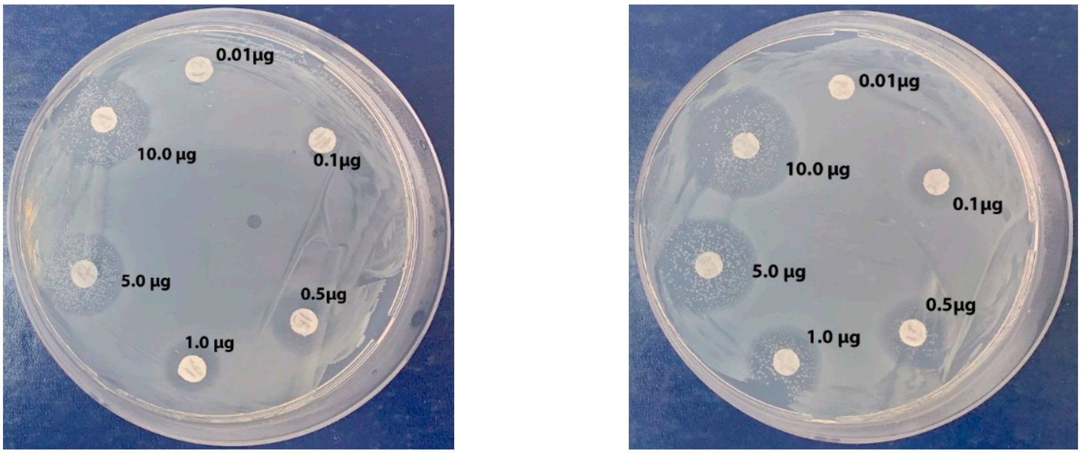

Figure S5. Doze-dependent inhibition of the growth of multi-drug resistant clinical isolates of *Klebsiella pneumoniae* with Bialaphos (left, µg of tripeptide per disk) and *L*-Leu-*L*-PT (right, µg of dipeptide per disk).

Table S1. Inhibition of the growth of clinical isolates of multi-drug resistant Klebsiella pneumoniae (marked light ocher) and reference strain ATCC 13883 (marked Mauve) with Bialaphos, *L*-Leu-*L*-PT and antibiotics of different classes.

	**Antibiotics and PT derivatives**	**Strain 1161**	**Strain 1158**	**Strain 1133**	**ATCC 13883**
		Diameter of growth zones, mm*⁾			
1.	Amikacin, 30 µg/disk	0/0	0/0	11/9	23/23
2.	Ampicillin, 10 µg/disk	0/0	0/0	0/0	0/0
3.	Gentamicin, 10 µg/disk	0/0	0/0	19/16	23/24
4.	Ciprofloxacin, 5 µg/disk	0/0	0/0	0/0	33/35
5.	Cefazolin, 30 µg/disk	0/0	0/0	0/0	22/24
6.	Tetracycline, 30 µg/disk	22/22	0/0	0/0	19/20
13.	Bialaphos, 5 µg/disk	16/15	16/16	17/16	18/22
14.	*L*-Leu-*L*-PT, 5 µg/disk	18/17	18/17	18/20	18/18
7.	Cefotaxime, 30 µg/disk	0/0	0/0	0/0	40/42
8.	Levofloxacin, 5 µg/disk	0/0	0/0	0/0	32/34
9.	Polymyxin B, 300 IU/disk	12/10	13/14	14/14	16/18
10.	Tobramycin, 10 µg/disk	0/0	0/0	0/0	21/20
11.	Trimethoprim, 5 µg/disk	0/0	0/0	0/0	18/21
12.	Ampicillin/Sulbactam, 10/10 µg/disk	0/0	0/0	0/0	13/15
13.	Bialaphos, 5 µg/disk	15/14	17/19	17/18	21/23
14.	*L*-Leu-*L*-PT, 5 µg/disk	17/17	16/19	21/21	19/22

*⁾ Dish 1/dish 2 (one of two dishes is depicted at Figure 3, for ATCC 13882, and at Figure 4 for multi-drug resistant strains 1161, 1158 and 1133).
*⁾ Light ocher section of the Table is illustrated by Figure 4; mauve section – by Figure 3.

Table S2. Multi-drug resistant clinical isolates of *Klebsiella pneumoniae*. R – resistant; S – sensitive; Antibiotics verified in this study are in the left column and are in blue.

**Antibiotic**	***K.pneumoniae* strain**			**Antibiotic**	***K.pneumoniae* strain**		
	**1161**	**1158**	**1133**		**1161**	**1158**	**1133**
Amikacin	R	R	R	Amoxicillin/clavulanic acid	R	R	R
Ampicillin/Sulbactam	R	R	R	Aztreonam	R	R	R
Ampicillin	R	R	R	Cefepime	R	R	R
Cefazolin	R	R	R	Cefoxitin	R	R	R
Cefotaxime	R	R	R	Ceftazidime	R	R	R
Ciprofloxacin	R	R	R	Ceftriaxone	R	R	R
Gentamicin	R	R	S	Cefuroxime	R	R	R
Levofloxacin	R	R	R	Ertapenem	R	R	R
Tetracycline	S	R	R	Imipenem	R	R	R
Tobramycin	R	R	R	Meropenem	R	R	R
Trimethoprim/sulfamethoxazole	R	R	R	Piperacillin	R	R	R
Polymyxin B	S	S	S	Piperacillin/tazobactam	R	R	R
				Tigecycline	S	S	R

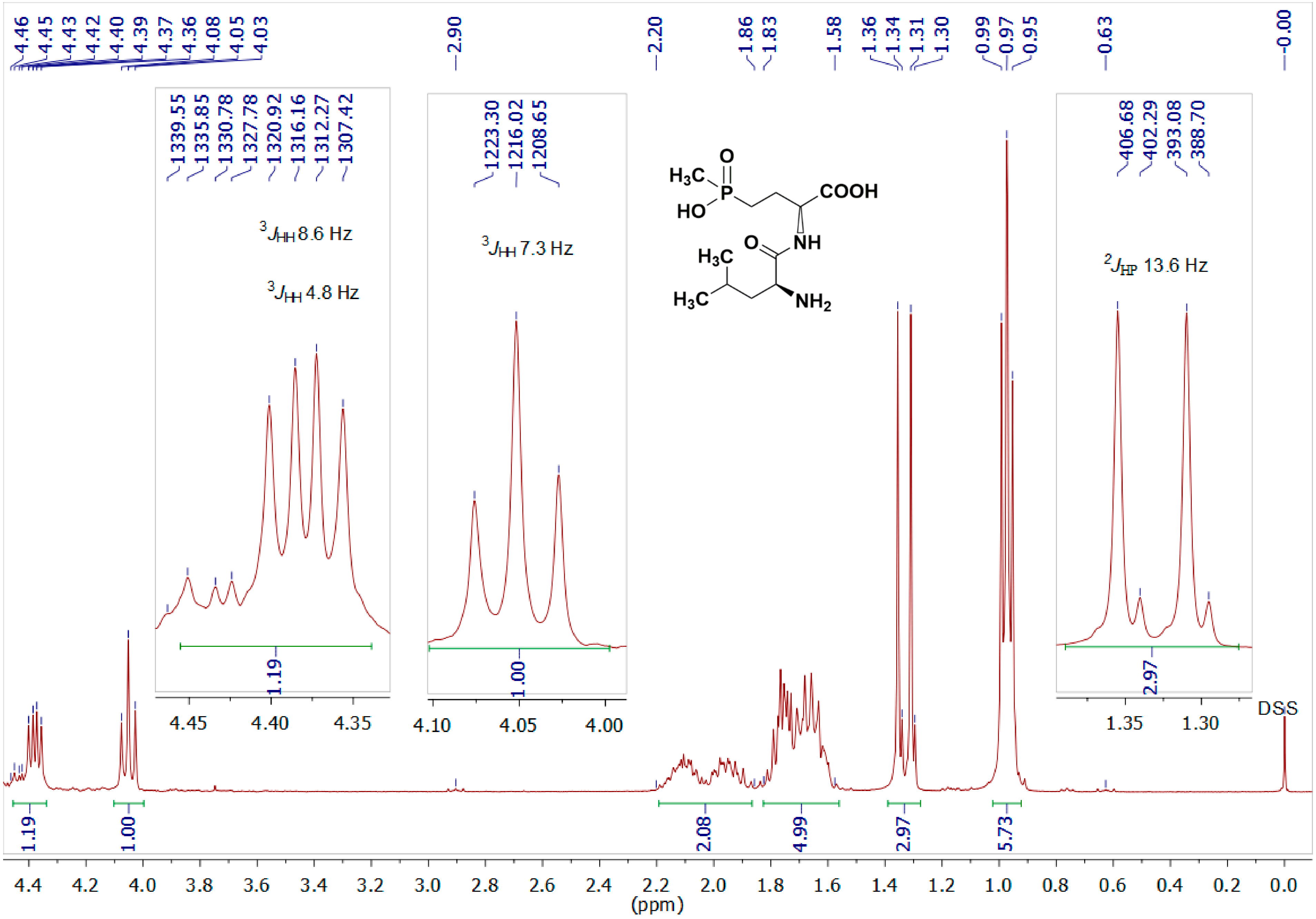

Figure S6. ^{1}H-NMR spectrum of *L*-Leu-*D*-PT with about 10% of *L*,*L*-diastereomer (fraction n. 7).

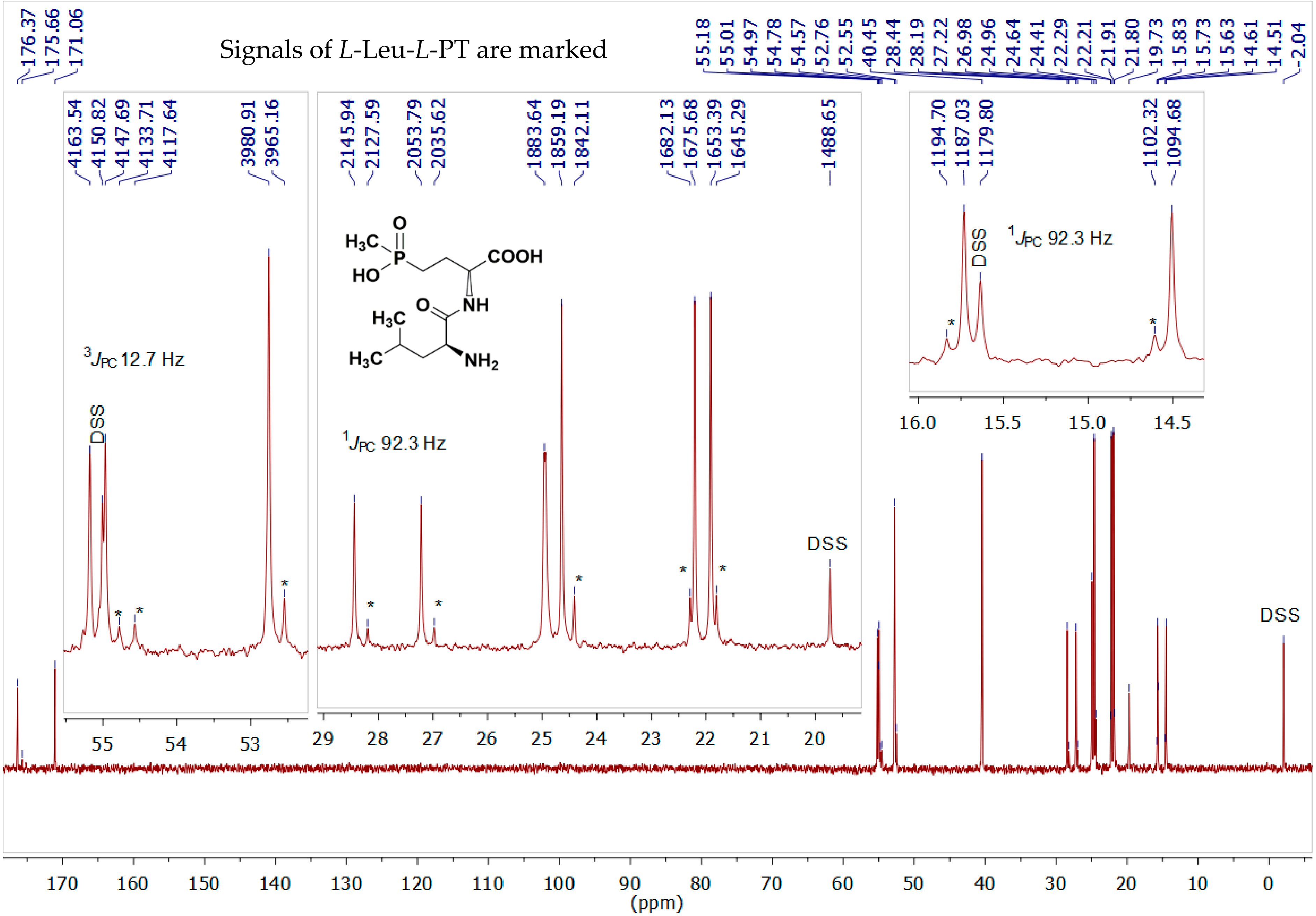

Figure S7. ^{13}C-NMR spectrum of *L*-Leu-*D*-PT with about 10% of *L,L*-diastereomer (fraction n. 7).

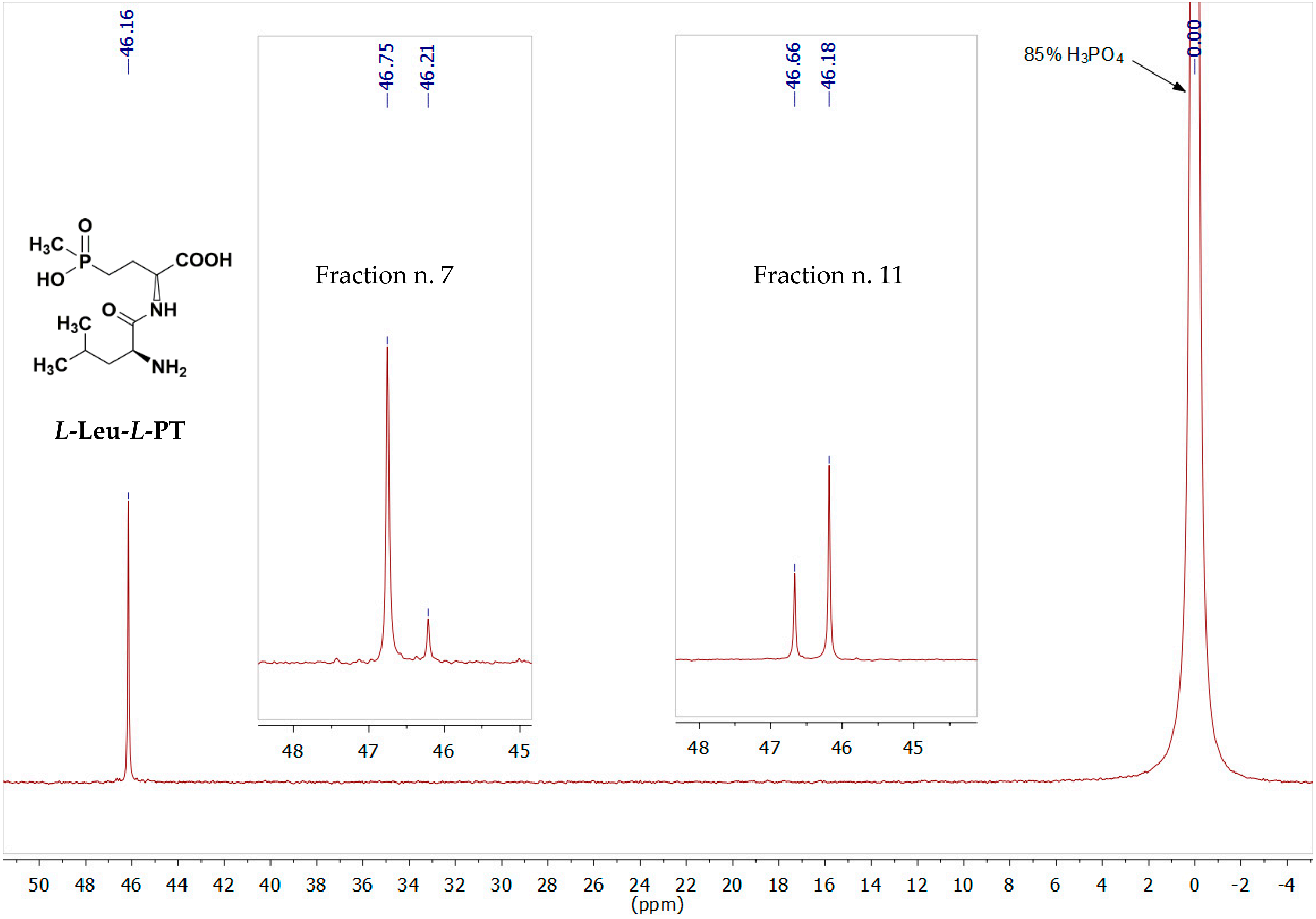

Figure S8. Combined ^{31}P-NMR spectra of *L*-Leu-*L*-PT, *L*-Leu-*D*-PT with about 10% of *L,L*-diastereomer (fraction n. 7) and *L*-Leu-*rac*-PT (fraction 11).

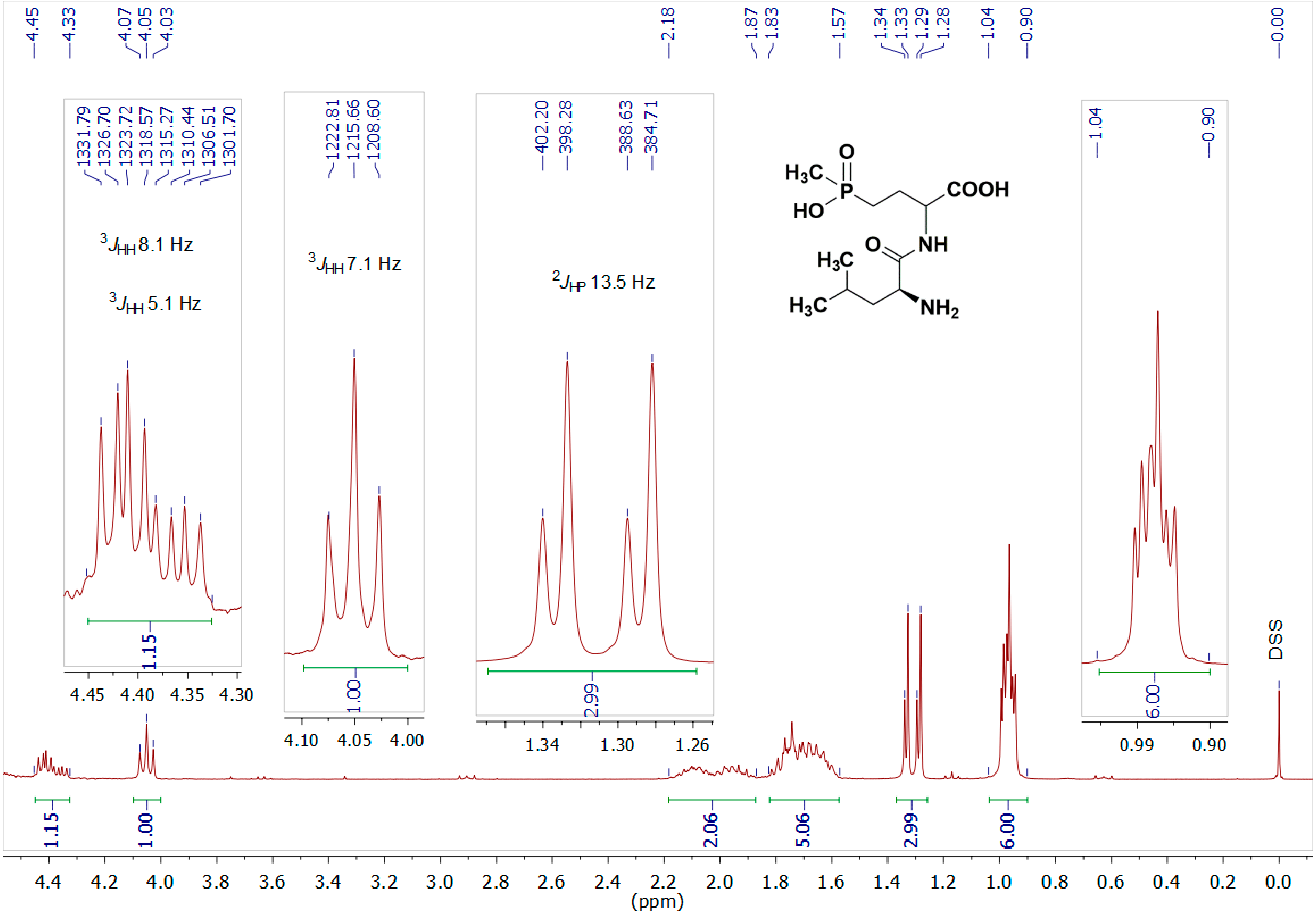

Figure S9. ^{1}H-NMR spectrum of *L*-Leu-*rac*-PT (fraction n. 11).

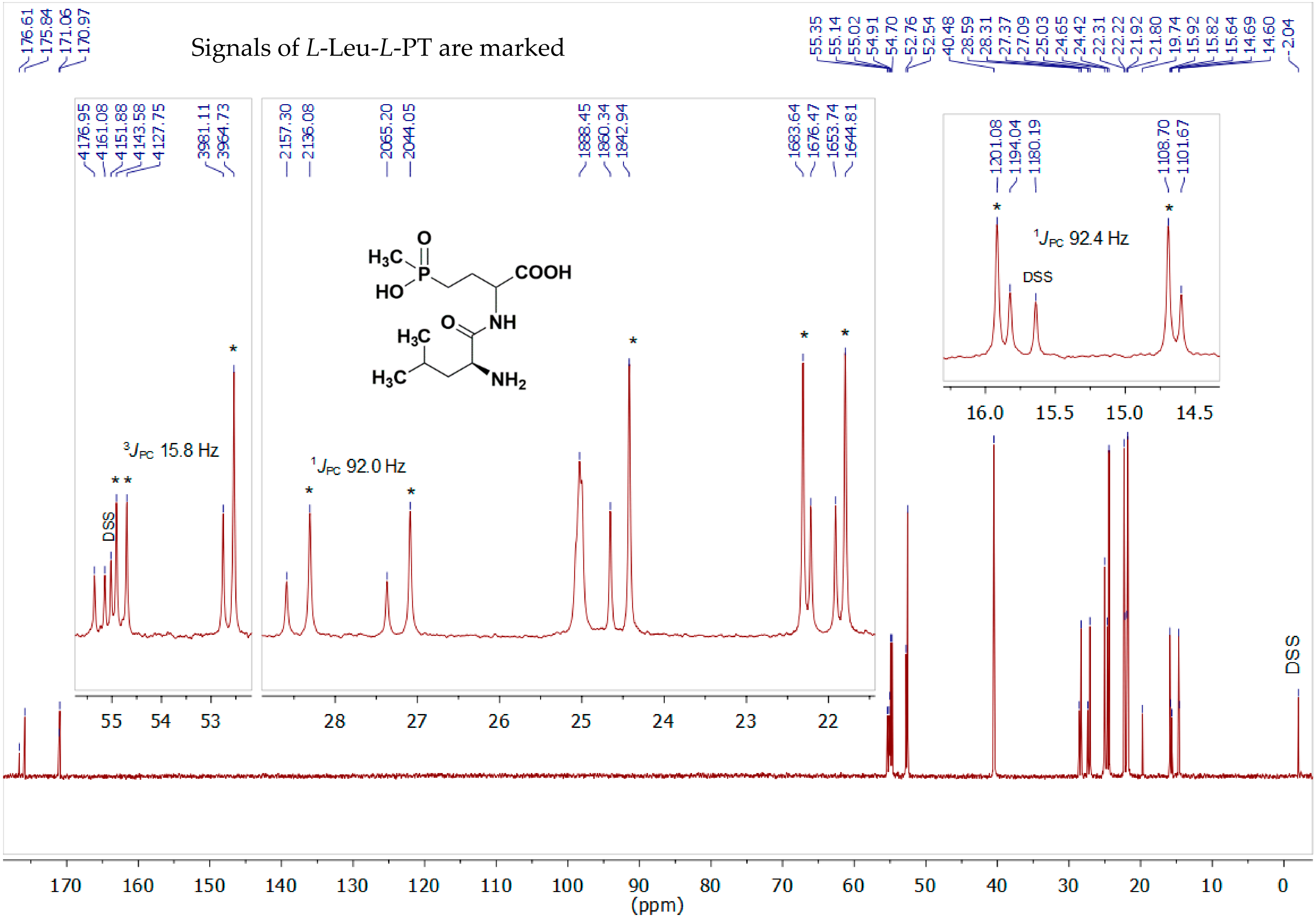

Figure S10. ^{13}C-NMR spectrum of *L*-Leu-*rac*-PT (fraction n. 11).

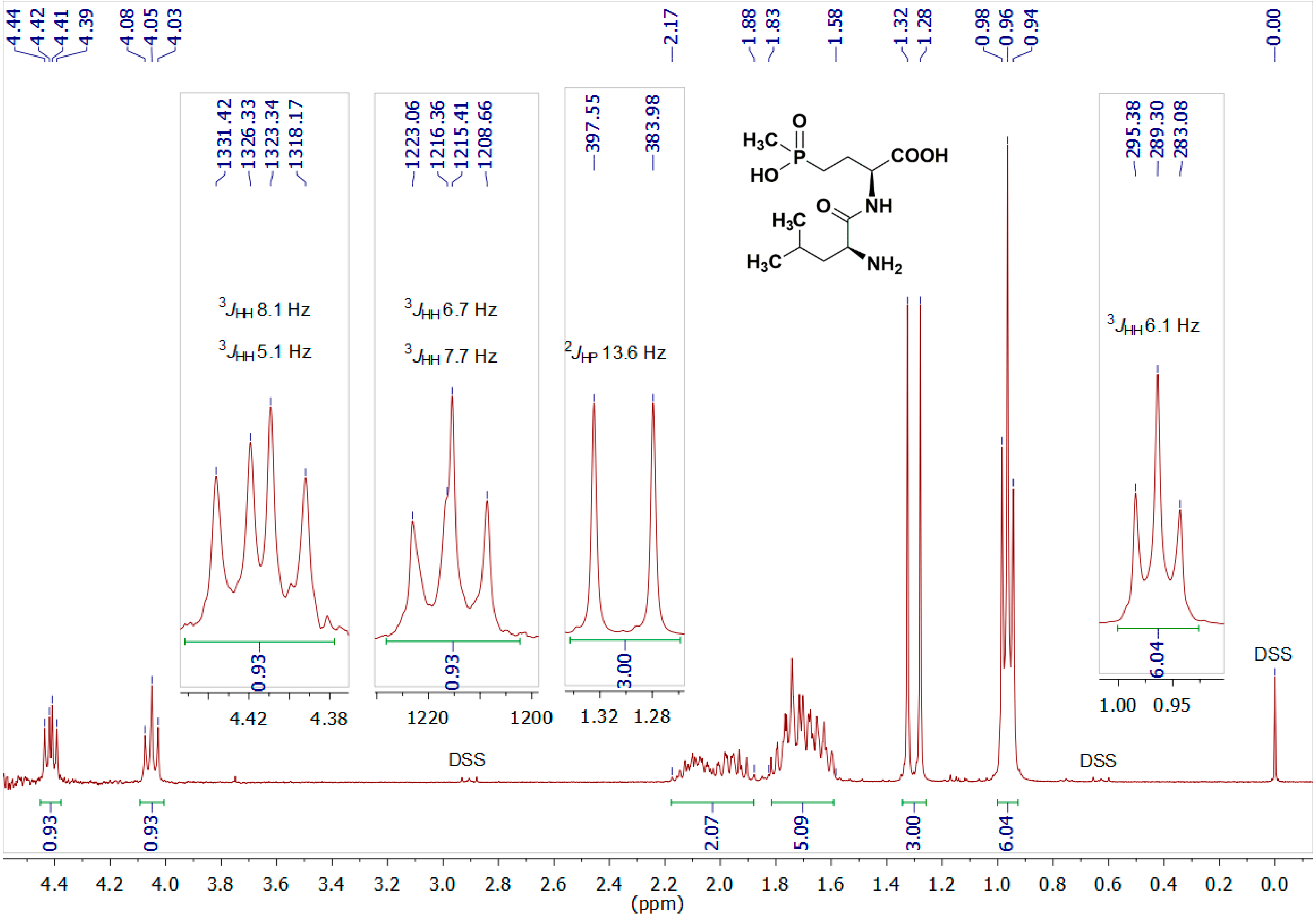

Figure S11. ^{1}H-NMR spectrum of *L*-Leu-*L*-PT.

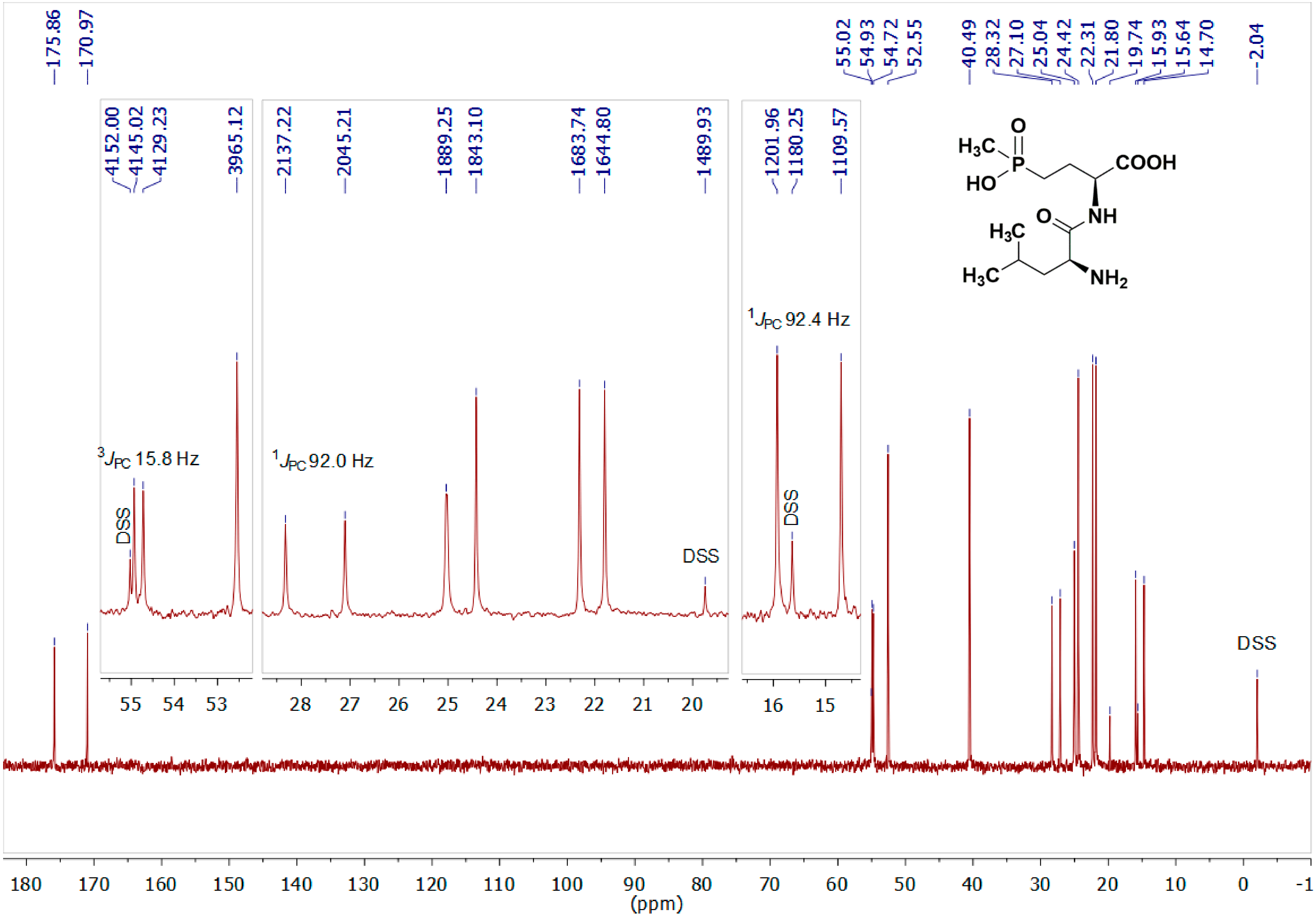

Figure S12. ^{13}C-NMR spectrum of *L*-Leu-*L*-PT.
